# Heterogeneous and interactive effects of payments for ecosystem services on household income across giant panda nature reserves

**DOI:** 10.1016/j.heliyon.2024.e34866

**Published:** 2024-07-18

**Authors:** Youqi Zhang, Yujun Wang, Hongbo Yang, Vanessa Hull, Jindong Zhang, Fang Wang, Zhiqiang Zhao, Jianguo Liu

**Affiliations:** a Key Laboratory of Southwest China Wildlife Resources Conservation, China West Normal University, Ministry of Education, Nanchong, Sichuan Province, 637009, China; b Research Center for Eco-Environmental Sciences, Chinese Academy of Sciences, Beijing, 100085, China; c Department of Wildlife Ecology and Conservation, University of Florida, Gainesville, FL, 3261 1, USA; d Ministry of Education Key Laboratory for Biodiversity Science and Ecological Engineering, Coastal Ecosystems Research Station of the Yangtze River Estuary, Institute of Biodiversity Science, School of Life Sciences, Fudan University, Shanghai, 200438, China; e Center for Systems Integration and Sustainability, Department of Fisheries and Wildlife, Michigan State University, East Lansing, MI, 48823, USA

**Keywords:** Natural forest conservation program (NFCP), Grain to Green program (GTGP), Interaction, Giant pandas' nature reserves, Sustainable development goals (SDGs)

## Abstract

Numerous Payments for Ecosystem Services (PES) programs have been implemented simultaneously around the world but their outcomes in the literature are not consistent and their interactive effects remain understudied. The Natural Forest Conservation Program (NFCP) and Grain to Green Program (GTGP) are two largest PES programs in the world, and many studies have evaluated their effects on household income. However, the identified effects often varied across different studies and the factors explaining this variation are poorly understood. This study used linear regression and geographic detector analysis, based on questionnaire survey data from 14 giant panda natural reserves (NRs) in southwestern China, to evaluate the effects of the NFCP and GTGP on household income and the factors which moderate these effects. The results revealed that the effects of two PES programs on household income were spatially heterogeneous and enhanced by each other and livelihood activities, suggesting a synergistic interaction between policies and livelihood activities, particularly tourism. This study also found that livelihoods activities (e.g., labor migration and tourism), household capital (i.e., house area and farmland area) and demographic factors (i.e., number of labor and non-labor members), exhibit spatial heterogeneity in their effects on household income across NRs. These findings underscore the importance of considering local socioeconomic conditions and the interaction between policy and socio-economic conditions in PES program design to achieve desired outcomes, providing insights for policymakers and practitioners worldwide.

## 1 Introduction

The conflict between socio-economic development and ecological protection is an ongoing global problem that needs to be urgently addressed. Payments for Ecosystem Services (PES) are an important policy mechanism for co-regulating socio-economic development and ecosystem conservation [1]. Around 550 PES programs are being implemented worldwide, with an annual investment of approximately $36 billion [2]. Implementation of PES has been one of the main means to achieve a “win-win” situation for protection and development [3].

Given the substantial annual investment in PES worldwide, there is a great interest in evaluating and understanding the outcomes of PES programs [4]. Moreover, evaluating the effectiveness of PES in terms of ecological, economic, and social dimensions, and identifying the factors influencing these outcomes, can inform the design and implementation of PES programs to maximize desired benefits [5]. Therefore, many studies have evaluated the ecological, economic, and social outcome of PES cases. These studies focused on social-ecological changes after the implementation of PES programs [6,7], cost-effectiveness analysis of PES programs [8,9], the effect of PES programs on household incomes and livelihood patterns [10,11], as well as effect of PES programs on human well-being [3,12].

China is one of the most extensively implemented countries in the world for PES [4], initiating the largest two PES programs globally the Natural Forest Conservation Program (NFCP) and the Grain to Green Program (GTGP) [13]. The NFCP is mainly designed to protect forest resources by prohibiting deforestation while providing payment to enterprises for afforestation and to residents for patrolling and guarding forests. By providing grain or economic compensation to participating residents, the GTGP converts residents’ farmland with a slope of more than 15° or 25° to forest or grassland to enhance ecosystems [13,14]. Many studies that assessed the ecological benefits of two PES programs, including forest recovery [15], wildlife habitat restoration [16], and ecosystem services improvement [6]. Conversely, numerous studies have evaluated the socioeconomic outcomes of PES implementation and have identified inconsistent outcomes [[17], [18], [19], [20]]. Therefore, further researches are imperative to examine the socio-economic outcome of policies implementation across regions with varying environmental and socio-economic contexts, along with the factors influencing socio-economic outcomes of policies. Furthermore, prior researches predominantly emphasized the direct effects of policies and influencing factors on outcome variables, disregarding the interaction between policies and the socio-economic factors. Despite recent efforts to investigate the role of socioeconomic factors in policies effects [10,11], such study remains scarce.

**Fig. 1:**
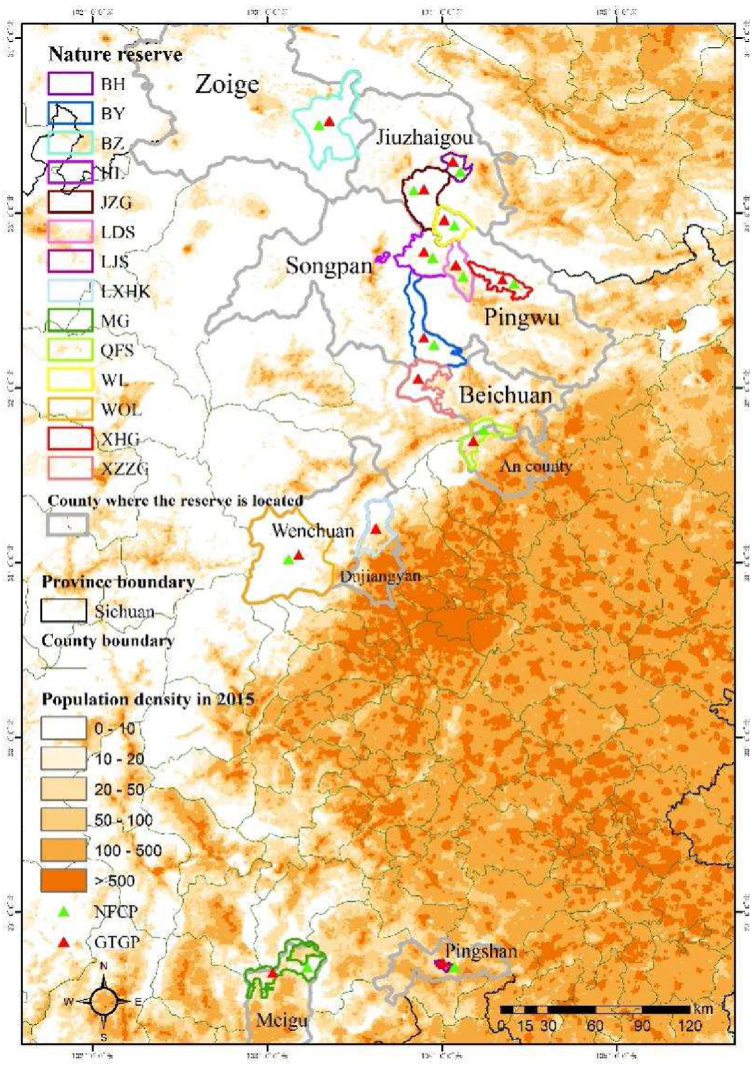
Geographical distribution of the 14 giant panda NRs included in this study. Note: The map shows the geographical location and county of 14 NRs, and the population density distribution information in 2015. For full names of NRs, please see Table 1.

To understand the complex effects of the NFCP and GTGP on rural household income and how these effects are moderated by other socioeconomic factors (e.g., livelihood activities), we conducted a questionnaire survey of communities living in and around 14 giant panda nature reserves (NRs) (Fig. 1). And then the linear regression and geographic detector analysis was used to evaluated the effects of the NFCP and GTGP on household income, as well as the interactions between NFCP, GTGP and socioeconomic factors. The main tasks of this study are as follows: 1) to evaluate the effects of two PES programs on household income, both at the overall 14 NRs scale and at the individual NR scale; 2) to assess the interactions between the two PES programs, as well as between PES programs and household socio-economic factors, on household income; 3) to identify critical factors that contribute to the significant and positive economic outcomes resulting from the implementation of the two PES. The findings of this study could provide crucial insights into understanding the outcomes of PES implementation, while informing PES decisions in China and beyond to maximize their desired benefits. Furthermore, this study is conducted in NRs where the conflict between biodiversity conservation and socio-economic development of human-beings poses significant challenges, making the findings hold practical significance for leveraging PES to strike a balance between biodiversity conservation and local development.

## 2 Literature review

The effects of GTGP and NFCP on the economic income have attracted much attention from the Chinese government and researchers. Numerous studies have assessed the effect of these two policies on residents' economic income, but the conclusions of different studies are diverse and even contradictory. Some investigations showed that GTGP had positive effects on residents' income. For example, Liu analyzed a panel dataset that covers 1921 households in 6 provinces for 14 consecutive years (1995–2008) and found that the GTGP had a positive impact on farmers’ income [21]. Another empirical study, based on data of 952 households sampled from 6 provinces from 1995 to 2016, found that the GTGP had increased the land-based permanent income, off-farm permanent income, and off-farm transitory income of rural households by 4.66 %, 2.05 % and 2.98 %, respectively [22]. Gao and Jin also found that the latest round of GTGP positively impacted rural livelihoods regardless of whether the compensation is included in livelihood estimation itself [23]. In contrast, some studies showed that the GTGP only had a significant positive effect on the property income of farmers, but the effect on overall income was not evident and even negative [17,18]. Similar to GTGP, the economic effect of NFCP implementation has been mixed in existing researches due to differences in study areas, timescales, and research methodologies. Some studies indicated that NFCP reduced farmers' income [19,24,25], while others showed that the implementation of NFCP increased farmers' income [20,26]. While these studies offer valuable insights into the outcome of PES implementation, they have mainly concentrated on the direct effects of PES, overlooking the complexity of effects, such as interactions between different PES programs, interactions between policy and non-policy factors, and the pathways through which these effects manifest.

Recently, increasing studies has focused the complexity of PES effects. Wang et al. [27] and Yuan et al. [28] found that ecological compensation policies can indirectly promote residents' sustainable livelihood strategy through an interaction with livelihood capital. Yang et al. found that NFCP and GTGP payment negatively affected total household income when examined separately, but jointly led to a positive effect [29]. Wu et al. found the total effect of the GTGP on household income is not statistically significant, but the GTGP significantly increased the participations in local non-farm jobs, which leads to increased household incomes [11]. Yang et al. also found that GTGP can promote residents to participate in tourism and migrant work to increase household income, but this can only offset 34 % of the income loss due to reduced crop production, while policy subsidies can only offset 11 % of the loss, which amounts to an overall negative impact on residents' income [10]. Despite these studies, studies that evaluated the interactions between PES programs and between PES and household socioeconomic factors are still scarce, which limits our understanding of how the effects of PES programs are moderated by other PES programs and diverse factors across different contexts. Therefore, further studies are needed to clarify the effect of PES policies and other factors (e.g., households' livelihoods) on residents’ economic income and the interaction effects of multiple PES programs (i.e., NFCP and GTGP) and household livelihoods.

## 3 Material and methods

### 3.1 Study area

In this study, we selected 14 giant panda NRs, including 8 national NRs, 4 provincial NRs, and 2 county-level NRs, including Baihe, Baiyang, Baozuo, Huanglong, Jiuzhaigou, Laojunshan, Longdishui, Longxihongkou, Meigu, Qianfoshan, Wanglang, Wolong, Xiaohegou, Xiaozhaizigou (Fig. 1). The counties where the reserves are located, including Pingwu, Beichuan, Wenchuan, Jiuzhaigou, Songpan, Ruoergai, Meigu, An county, Pingshan and Dujiangyan, were all classified as poverty-stricken in 2015 by the government.

The 14 NRs are distributed across Minshan, Liangshan, and Qionglai mountain ranges. These NRs collectively cover 376,824 hm^2^ of giant panda habitat, accounting for 27.9 % of the entire giant panda habitat in Sichuan Province. The total number of giant pandas in those NRs is 388, accounting for 35.8 % of the population in Sichuan province. The population of residents living in and around NRs ranges from 2726 to 23,368 people. The income status of local residents also varies greatly across different reserves (Table S1), with per capita annual income ranges from 1013 RMB to 9826 RMB (1 RMB equated to roughly 0.1592 USD in 2015, Table S1).

**Table 1:** Linear regression results for the effects of various factors on household income in different NRs.

Parameter	14NRs	Baihe	Baiyang	Baozuo	Huanglong	Jiuzhaigou	Laojunshan	Longdishui
Constant	3.402(0.060)***	3.588(0.235)***	3.202(0.167)***	3.223(0.211)***	3.605(0.258)***	3.891(0.227)***	3.212(0.173)***	2.950(0.442)***
**Household capital**								
House area (in m^2^)	0.001(0.000)***	0.001(0.000)	0.000(0.000)	0.001(0.1)**	−0.001(0.000)*	0.001(0.000)	0.000(0.001)	0.001(0.001)
Farmland (in mu)	0.00(0.001)	−0.029(0.027)	0.000(0.000)	−0.002(0.007)	−0.004(0.012)	0.006(0.035)	0.015(0.011)	−0.007(0.005)
**Demographic factors**								
Non-labor	0.026(0.011)*	0.020(0.046)	0.061(0.035)	0.061(0.028)*	−0.063(0.071)	−0.048(0.042)	−0.020(0.031)	0.090(0.086)
Labor	0.041(0.012)***	−0.013(0.035)	0.073(0.034)*	0.002(0.030)	0.073(0.045)	0.010(0.044)	0.075(0.037)*	−0.022(0.075)
**Livelihood activities**								
Migrant	0.152(0.034)***	0.304(0.152)*	0.286(0.097)**	0.121(0.098)	0.131(0.193)	0.100(0.154)	0.602(0.110)***	0.203(0.189)
Tourism	0.621(0.046)***	0.569(0.178)**	0.157(0.174)	0.352(0.134)*	0.712(0.186)***	0.804(0.116)***	0.775(0.293)**	0.334(0.254)
Agriculture	0.159(0.031)***	0.141(0.115)	0.255(0.085)**	0.359(0.086)***	0.448(0.307)	Na	0.105(0.106)	0.524(0.281)
Graze	0.210(0.044)***	0.459(0.164)**	0.269(0.109)*	0.556(0.110)***	0.546(0.229)*	−0.040(0.339)	−0.076(0.147)	−0.113(0.450)
Local non-farm	0.296(0.032)***	0.489(0.138)**	0.328(0.089)***	0.483(0.105)***	0.106(0.186)	0.321(0.133)*	0.380(0.098)***	0.345(0.203)
**Policy participation**								
GTGP	0.049(0.042)	−0.017(0.183)	0.084(0.106)	0.041(0.166)	0.477(0.200)*	0.145(0.162)	−0.036(0.096)	0.240(0.362)
NFCP	0.165(0.031)***	0.070(0.115)	0.100(0.092)	0.001(0.089)	0.008(0.167)	−0.173(0.185)	0.232(0.094)*	0.617(0.176)**
Adjusted R^2^	0.242	0.214	0.287	0.349	0.245	0.423	0.290	0.294

Parameter	Longxihongkou	Meigu	Qianfoshan	Wanglang	Wolong	Xiaohegou	Xiaozhaizigou
Constant	3.631(0.192)***	2.658(0.303)***	3.222(0.329)***	3.825(0.423)***	3.164(0.342)***	2.685(0.289)***	2.760(0.416)***
**Household capital**							
House area (in m^2^)	0.001(0.001)	0.004(0.001)**	0.000(0.000)	0.002(0.001)	0.001(0.000)**	0.001(0.001)	0.000(0.001)
Farmland (in mu)	0.052(0.019)**	−0.006(0.014)	0.009(0.016)	−0.002(0.016)	0.003(0.009)	0.002(0.015)	0.004(0.007)
**Demographic factors**							
Non-labor	−0.042(0.055)	0.037(0.051)	0.104(0.041)*	0.018(0.069)	0.079(0.027)**	0.102(0.051)	0.072(0.059)
Labor	0.054(0.038)	0.118(0.061)	0.071(0.050)	−0.056(0.066)	0.016(0.034)	0.121(0.056)*	0.095(0.064)
**Livelihood activities**							
Migrant	0.009(0.123)	0.439(0.265)	0.189(0.117)	−0.104(0.204)	0.152(0.082)	0.300(0.122)*	0.149(0.139)
Tourism	0.137(0.182)	0.665(0.228)**	0.454(0.203)*	0.280(0.248)	0.269(0.110)*	1.073(0.408)*	0.461(0.205)*
Agriculture	−0.009(0.140)	0.141(0.195)	0.115(0.148)	0.597(0.204)**	0.187(0.083)*	0.139(0.139)	0.334(0.144)*
Graze	0.243(0.241)	0.338(0.173)	0.313(0.285)	0.334(0.267)	0.194(0.101)	0.077(0.145)	0.161(0.155)
Local non-farm	0.351(0.133)*	0.175(0.143)	0.558(0.113)***	0.259(0.184)	−0.038(0.187)	0.444(0.111)***	0.594(0.143)***
**Policy participation**							
GTGP	−0.039(0.156)	0.084(0.177)	−0.314(0.281)	−0.048(0.334)	0.025(0.147)	0.218(0.154)	0.124(0.326)
NFCP	–	−0.100(0.254)	0.164(0.124)	0.041(0.180)	0.520(0.323)	0.334(0.165)*	–
Adjusted R^2^	0.171	0.287	0.347	0.217	0.215	0.461	0.224

Note: the data shown in the table are regression coefficients and standard errors of the models; Marks***, **, *, indicate significance at the 0.1 %, 1 %, and 5 % level, respectively. “0” means that the coefficient of this parameter is small and the contribution to the model is small; “Na” means that this parameter is excluded from the model when the model is established; “-” indicates that no one under investigation was involved in the policy project. Definitions of variables are shown in Table S1.

### 3.2 Household data collection

Our data collection was conducted at the household level, like many of previous studies [1,9,10]. Between July and August 2015, we collected the household data in 14 giant panda reserves and their surrounding areas through in-person household surveys. Considering that the head of the household (typically male in these regions) and his wife are the main decision-makers of family activities and are familiar with family affairs, they were selected as the interviewees. Initially, we identified the village closest to the reserve with an administrative map, and then visited the households from door to door. The survey started with households located on the two sides of the main road in the village, gradually extending to those positioned on hillsides, given the scattered nature of houses in our survey areas along hillsides. Whenever the head of the household was available, we conducted a questionnaire survey, obtaining verbal consent from the interviewees before formally initiating the interviews. In addition, to ensure the consistency and validity of each acquired survey data, we provided comprehensive training to all interviewers prior to the surveys. Finally, we obtained a total of 1336 valid questionnaires, with the sample size from each individual reserve exceeding 50, representing over 20 % of total households in each surveyed village. Almost all of the villagers were cooperative, resulting in a survey response rate exceeding 99 %. Through the household survey, we collected information on demographic, assets (house area and farmland area), livelihood activities (in five categories including agriculture, grazing, tourism, migrant work and other local non-agricultural activity), income, expenditure, and participation in PES policies (see Tables S1 and 3 for a description of variables). The category of other local non-agricultural activities could include roles as local governmental staff or temporary jobs in reconstruction in the period after a major earthquake in the region in 2008. Ethical approval for our data collection procedures was granted by the Institutional Review Board of Michigan State University (Approval Number: 10–660).

### 3.3 Data analysis

#### 3.3.1 Evaluating the effects of PES on household income by linear regression

We used linear regression analysis (SPSS 22.0) to evaluate the effect of NFCP, GTGP and other livelihood factors on the economic income of residents in all reserves together and in each individual reserve separately. Since the objective of this study was to investigate the complex effects of PES programs in the context of different factors (i.e., multiple policies, household characteristics), rather than quantify the effects of each PES individually. Therefore, we choose the regression method rather than more rigorous tools (e.g., matching, differences-in-differences) used in other studies [8,30] for this analysis. And the indicators of regression analysis are selected based on previous studies [8,11,18,24,[28], [29], [30]] and shown in Table S1.

#### 3.3.2 Evaluating the interactions among PES and livelihoods by geographic detector

The Geographic Detector, a relatively new method that reveals the driving force behind the effects, was used to analyze which factors have the greatest effect on resident income, whether there is a significant difference in the economic income of residents with different policy participation status, and the interaction between policies and livelihood activities on household income. This method does not need to consider the multicollinearity between the variables and can measure the spatially stratified heterogeneity and influencing factors [31,32]. Geographic detector can analyze qualitative data and can detect the interaction between two variables, as well as direction, and whether the relationship is linear or non-linear. It has been widely used in numerous research fields such as disease factor assessment [33], social economy [34], soil erosion [35], and vegetation coverage [36]. Specifically, we selected PES participation (participation versus non-participation) and the factors affecting household income with respect to five livelihood types (migrant work, tourism, agricultural labor, grazing animals, local non-farm work) as independent variable Xi, and used the Geographic Detector for quantitative analysis. Then, the risk detector of Geographic Detector was used to distinguish between groups of households that affected and did not affect in PES, and examined whether there is a significant difference between the mean income of the two groups of households (p < 0.05). Its formula is shown in Wang et al. [31]. The factor detector was used to analyze which factors have the greatest effect on resident income. It detects spatially stratified heterogeneity of variable Y (household income in this study), and impacts of different factors (Xi) on the variable Y, and analyses the contribution of each influence factor. The degree of the influence is measured by *m*, whose value range is between 0 and 1. The greater the *m* value, the greater the influence of this factor on household income. The interaction detector is used to detect the interaction among GTGP and NFCP, among policies and livelihood activities and between any two livelihood activities. By comparing the effect of single factors on variable Y and effects of interactions between two factors on variable Y, we identified whether there are interactive effects. We then determined the interaction type (e.g., nonlinear weakening, single-factor nonlinear weakening, two-factor enhancing, independent, nonlinear enhancing). In the interaction detector, *m* can also represent the magnitude of influence of the interaction. Definitions of different types of interaction effects are shown in Fig. S1. The specific method and formula can also be found on the website (http://www.geodetector.cn/).

#### 3.3.3 Comparative analysis of the characteristics of affected and unaffected households

Taking the 14 NRs as a whole, we used T-test to compare and test the main household characteristics of the family group (such as laborers, house area) with and without the significant effect of the PES programs, and to further analyzed the reasons for the different economic effects of policies on different families.

## 4 Results

### 4.1 Effects of PES and livelihood activities on household income

At the overall scale of 14 NRs, house area, demographic factors, participation in the NFCP and all livelihood activities had a significant positive effect on household income (Table 1). At the individual reserve scale, participation in the NFCP had a significant positive effect on household income in Laojunshan, Longdishui and Xiaohegou Nature Reserves, while participation in the GTGP only had a significant positive effect on income of households in Huanglong Nature Reserve. The effects of these policies on household income in the other 10 NRs were not significant. Participation in livelihood activities had a significant positive effect on household income in many, but not all reserves. The number of non-laborers, such as students (∼16 years old) and elderly (65 years old ∼), and the number of laborers and house area all had a significant positive effect on household income in some reserves, including Baiyang, Baozuo, Huanglong, Laojunshan, Longxihongkou, Meigu, Qianfoshan, Wolong, Xiaohegou.

Among them, the adjusted linear model of Xiaohegou and Jiuzhaigou Nature Reserve has a relatively higher R^2^ of 0.461 and 0.423; followed by Qianfoshan, Longdishui, Laojunshan, Meigu, Baiyang, Huanglong, Xiaozhaizigou, Wanglang, Wolong and Baihe Nature Reserve, with the adjusted R^2^ of 0.349, 0.347, 0.294, 0.290, 0.287, 0.287, 0.245, 0.224, 0.217, 0.215 and 0.214, the smallest R^2^ is in Longxihongkou Nature Reserve, which is 0.171. All linear models were statistically significant (*p* < 0.01), indicating that the results were not generated by random processes (Table 1).

### 4.2 Main factors influencing household income in different reserves

The risk detector analysis showed that across 14 NRs pooled, household income of residents who participated in the GTGP was significantly higher than that of households that did not participate in the GTGP (Table 2). However, there was no significant difference in household income of residents who participated in the NFCP and those who did not participate, although the average income of the affected families was higher (Table 2).

**Table 2:** The income of households participating and not participating in the GTGP and NFCP.

	Non-participation	Participation	Statistical difference
**GTGP**	25748.17	34112.13	Y
**NFCP**	32648.37	33148.24	N

Note: The data shown in the table are the average household income; Y indicates significant difference at a 5 % level, and N indicates no significant difference at a 5 % level.

**Fig. 2:**
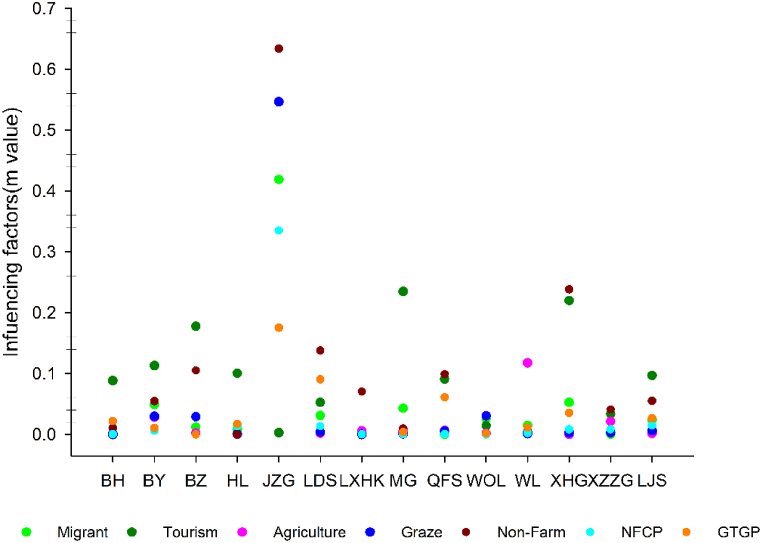
Factors (*m* value) influencing household income in each NR.

Through the results of factor detector analysis, we found that tourism had the greatest influence on household income relative to other livelihood activities in Baihe, Baiyang, Baozuo, Huanglong, Meigu and Laojunshan NRs. Local and other non-farm livelihood activities (e.g., doing odd jobs locally and running a small shop) were the most influential livelihood factor in Jiuzhaigou, Longdishui, Longxihongkou, Qianfoshan, Xiaohegou and Xiaozhaizigou NRs. The most influential livelihood factor was agriculture in Wanglang Nature Reserve and grazing in Wolong Nature Reserve (Fig. 2).

### 4.3 Interaction effects among PES and livelihoods on household income

The interaction between participation in the GTGP and NFCP on household income was significant and enhancing (*m*(GTGP∩NFCP) = 0.0024＞Max(*m*(GTGP), *m* (NFCP)), which means that the influence of participating in both policies on family income is greater than the sum of the singular effects of participating in each of the policies alone (Table 3). The interactions among policies and livelihood activities were also enhancing, as were the interactions among any two livelihood activities (Table S3). This result indicates that the income of households that participated in PES policy and any livelihood mode at the same time had an added positive effect relative to participation in only individual livelihood activities or PES policies. In particular, the interactions between tourism and other livelihood activities (migrant, agriculture, graze, non-farm) and policies were 0.094, 0.095, 0.094, 0.099, 0.093 and 0.094, which were far greater than the interaction effects among other influencing factors (Table 3).

**Table 3:** Interactions among 7 influencing factors on household income in 14 panda NRs.

	Migrant	Tourism	Agriculture	Graze	Non-Farm	GTGP	NFCP
**Migrant**	0						
**Tourism**	0.0938	0.0896					
**Agriculture**	0.0026	0.0950	0.0009				
**Graze**	0.0084	0.0937	0.0033	0.0007			
**Non-Farm**	0.0066	0.0994	0.0117	0.0075	0.0058		
**GTGP**	0.0033	0.0934	0.0031	0.0030	0.0090	0.0016	
**NFCP**	0.0027	0.0945	0.0054	0.0040	0.0115	0.0024	0

*See Table S1 for the definitions of the factors.

### 4.4 Differences among households affected and non-affected by PES

**Fig. 3:**
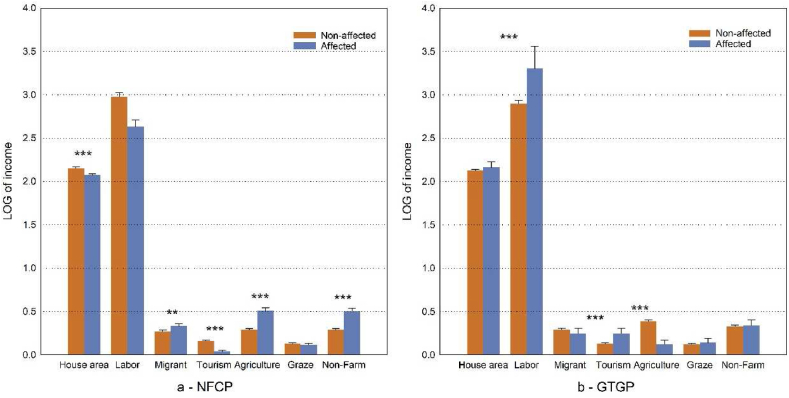
Comparison of income between groups of households affected and non-affected by (a) NFCP and (b) GTGP, respectively. Note: the data shown in the table are average values; Marks *** and **indicate the significance at 0.1 % and 1 % respectively. For convenience of display, the house area in the drawing is expressed as the value after LOG conversion.

Across the 14 NRs as a whole, the house area of households whose income was affected by NFCP was significantly smaller than that of households experiencing no NFCP effect on income. In terms of livelihood activities, the average probability of migrant work, agriculture and local non-agricultural livelihoods in NFCP-affected households was significantly higher than that of non-NFCP-affected households, while the average probability of tourism participation of NFCP-affected households was significantly lower than that of non-NFCP-affected families (Fig. 3a).The averages number of laborers in the households affected by GTGP was significantly higher than that in the non-affected households. In terms of livelihood activities, the average probability of tourism participation in households affected by GTGP was significantly higher than that in non-affected households, while the average probability of agricultural participation in households affected by GTGP was significantly lower than that of non -affected households (Fig. 3b).

## 5 Discussion

There is usually a strong spatial overlap between poverty-stricken areas and ecologically fragile areas in China and beyond [37]. Poverty-stricken areas are not only places where communities lack infrastructure and resources to meet livelihood needs, but also most areas that are of strategic importance for national and global ecological protection goals [38]. Ideally, policymakers expect PES programs (e.g., NFCP and GTGP) to have significant and positive economic effects in order to meet the intended policy goals. Our pooled regression analysis of all 14 NRs indeed showed that NFCP (but not GTGP) had positive effects on household income. But, the positive effects of NFCP and GTGP only presented in 3 and 1 reserves, when we test the relationship with household income one by one. These findings underscore the spatial heterogeneity characterizing the outcomes of PES implementation, aligning with previous researches [18]. Wang, Yang [18] also found that participation in the NFCP and GTGP had no effect on household income at the overall scale of the 30 NRs. However, at the scale of individual NRs, these two programs had no significant impact on residents' income in 88 % of NRs. At the overall scale, the GTGP's lack of significant effects may be due to several factors. First, the GTGP exhibit a range of positive and negative impacts depending on the context, which can cancel each other out in a single analysis [18]. Second, as prices and the cost of living have risen rapidly over time, the impact of the fixed payments from the GTGP, established in 2000, on household income may now be negligible [18]. These two theories are supported by the results of our separate modeling of each NR's effects on household income (Table 1).

At individual NRs scale, there could be two main reasons for various income outcomes of NFCP and GTGP in different NRs. For NFCP, on the one hand, some studies have shown that most of the residents of NRs participated in the NFCP as a way of community co-management [39]. That is, these households obtained economic subsidies through regular protection patrols and logging supervision in each natural protection forest area. On the other hand, the NFCP may lead to a decline in forestry-derived income of farmers (refers to the residents’ income from forest resources, for example, collecting Chinese medical herbs and bamboo shoots and livestock grazing in NFCP areas) [40,41]. For some households, policy subsidies are not enough to offset the reduced forestry income of the whole family [42,43], which has led to a decline in the overall household income. For example, 22 % of the household income in Xiaohegou comes from policy subsidies. The residents around Laojunshan and Longdishui rarely rely on forestry, and the implementation of the NFCP barely reduced their previous income sources. In Laojunshan and Longdishui, 63 % and 79 % of household income comes from local and migrant work. Although the subsidy amount accounted for only 5 % and 7 % of their income, the subsidy amounted to a direct increase in additional income, so the implementation of NFCP has a positive effect on them. By contrast, the average annual income of households in Meigu was very low (1625 yuan, equated to roughly 259 USD in 2015) and almost all of this comes from forestry income, accounting for 44 %, and the policy subsidy only accounts for 12 % of total income, which is not enough to offset the loss of forestry-derived income, so the effect of NFCP is negative.

For GTGP, the reduction of cultivated land caused by the GTGP can lead to a decrease in agricultural income and an increase in food expenses since they need to buy more food with the reduction of household agricultural production, while government subsidies and income from other livelihood sources such as participation in tourism or migrant work cannot completely offset the economic losses of policy participation [10]. For instance, some studies found that the current payments of GTGP are solely based on the amount of cropland afforested, the potential impact of GTGP on remaining croplands was not considered [30]. Thus, the GTGP may have whittled away the compensation for farmers and ultimately led to farmers' aversion to participating in future protection work [30]. Conversely, the reduction of cultivated land may indirectly bring more benefit to local residents due to generation of the spare labor force in households that were otherwise engaged in agriculture, and the spare laborers are more likely to participate in tourism, migrant work, and other higher-income livelihoods, resulting in higher household incomes than non-participating households [44]. For example, tourism in Wolong Nature Reserve is well developed, and local residents prefer to high-income non-agricultural livelihood after giving up farmland. In Wolong, 44 % of household income comes from tourism. In addition, differences in subsidies amounts across reserves could also explain variation. While administering our questionnaire, we found that subsidy standards were varied, for example, the compensation from GTGP in Wolong Nature Reserve was much higher than other reserves because the central and local government invested more financial subsidies into this famous reserve every year [38].

Our study showed that the effect of participating in two policies simultaneously on household income was greater than that of participating in only one policy, highlighting the synergistic effect of two PES programs on income outcomes. Past studies have shown farmers' willingness to participate in PES programs is a main factor influencing implementation [45]. Therefore, a positive experience with one program may encourage a resident to take up another. The potential for the two programs to work together is a key finding that supports synergistic development of a toolbox of PES programs in this economically disadvantaged region in the future. Furthermore, in line with prior research [10,11], the findings of this study underscored the significance of livelihood activities in shaping the economic outcomes of PES. Specifically, our results showed the effects of NFCP and GTGP on household income were also enhanced by participation in other livelihood activities, especially tourism involvement. Other studies have also highlighted the effects of livelihood activities such as tourism, labor migration and local non-farm employment on household income [10,11]. The varied livelihoods identified across different studies to convey the economic effect of PES programs also suggest the spatial heterogeneity of the mediating role of livelihood activities. Therefore, the synergistic nature of these livelihood opportunities is important to consider for future conservation efforts. In particular, tourism may be a way to realize the sustainable development of protected areas such as NRs, considering the potential for residents' economic benefits, social benefits and ecological benefits [23,46].

Our results also revealed that household factors can explain differences in the effect of PES program participation on household income. The NFCP has had a significantly greater positive effect on families with fewer assets (such as small house area) and those relying on farming. The size of the house area is often indicative of the overall economic situation of a family, no matter in a rural or urban area. A smaller house area is indicative of a poorer economic situation for the family, and thus the government's economic subsidies have a greater effect on household income. Policy subsidies are more likely to have a significant positive effect on the income of families participating in farming (a high time cost and high human capital investment and low economic return activity) and less on those participating in tourism (a comparably fast return and high-income activity). At the same time, we also found that the NFCP had a greater positive effect on the income of households who participate in migrant work and have local non-agricultural livelihoods.

The GTGP has been implemented in most of the panda reserves for a long time (many since 2000). After cultivated land is converted into forest land, the most obvious change is the reduction of agricultural production activities. Households will produce spare labor, and they may participate in high-income livelihood activities such as tourism and migrant work. This indicates that implementation of GTGP can indirectly promote economic development in those communities around panda reserve s [13]. Therefore, when analyzing the household characteristics of affected or non-affected families, the labor force in affected families is significantly higher than that of non-affected families. In addition, the affected families are less involved in agricultural livelihood activities and more involved in tourism livelihood activities. A comparative analysis of household characteristics indicates that policymakers and policy implementers should direct greater attention to areas and households with lower economic levels and less diversified livelihoods. These include, for instance, smaller house areas and greater reliance on farming. At the same time, encouragement and support should be extended to engagement in suitable, high-income livelihoods, such as tourism.

## 6 Implications, limitations and future directions

Our research can contribute to future policy design, implementation, and evaluation in two ways. On one hand, the economic benefits of policies demonstrate spatial heterogeneity. Large-scale studies might obscure the true benefits of policy implementation, impacting policymakers' assessments of the economic, social, and ecological merits of policies. Therefore, it's advisable that future research select appropriate scales, considering a blend of large and small scales to comprehensively evaluate the benefits of conservation policies. Meanwhile, devising targeted, refined, and differentiated implementation plans or programs tailored to the varying regional context are needed to optimize desire outcomes in a wide range of policies implementation regions. On the other hand, this study underscores the significance of various community characteristics (e.g., house area, household livelihood activities, and assets) and the interaction between policies and local livelihoods. It's recommended that scientists, policymakers, and conservationists take into account shifts in household income and livelihood needs and the interactions between policies and livelihood activities when devising and implementing conservation policies. For instance, policymakers could contemplate implementing tailored livelihood support programs for diverse regions and households to expedite the transition to sustainable livelihoods. Digital technologies and the economy have been found to have a profound impact on socio-economic development [47,48]and can be used as tools to facilitate livelihood transformation. Integrating these technologies into conservation policies can enhance the effectiveness and efficiency of policy implementation, helping communities transition to more sustainable and resilient livelihoods.

It is important to acknowledge the limitations of this study and outline future research directions that may contribute to improving subsequent work. Firstly, the study relies on cross-sectional data from in-person interviews conducted in 14 giant panda NRs, which limits our ability to capture temporal dynamics and long-term effects of PES programs on household income. Longitudinal data would provide a more comprehensive understanding of these impacts over time. Therefore, future research should incorporate longitudinal data to better understand the temporal dynamics and long-term effects of PES programs on household income. This would allow for the assessment of trends and changes over time, providing a more robust analysis of the effectiveness of PES programs. Secondly, the study examines the interactions between PES programs and household livelihood activities, but the complex and potentially confounding interactions between various socio-economic and environmental factors are not fully disentangled. Employing advanced statistical and econometric methods in the future studies, such as structural equation modeling (SEM), could better account for the complex interactions and potential confounding variables in the analysis of PES effects. Thirdly, while poverty alleviation stands as the core target among the Sustainable Development Goals (SDGs), it merely represents one facet of the broader spectrum of socioeconomic SDGs. Future research should also assess the broader socioeconomic effects of PES programs, including their effects on good health and well-being, energy transition and social equity. This holistic assessment would provide a more comprehensive evaluation of PES programs in achieving SDGs.

## 7 Conclusions

This study used in-person interviews data from residents in 14 giant panda NRs to investigated the heterogeneous and interactive effects of two of the most important environmental policies in China—the NFCP and the GTGP —on household income. We found that the NFCP positively impacts household income at the overall scale of 14 NRs, whereas the GTGP does not show consistent effects at this aggregate level. However, these effects vary greatly across different NRs, underscoring the spatial heterogeneity in PES outcomes. The mixed economic results of NFCP and GTGP can be attributed to variations in local community engagement, reliance on forestry, and the adequacy of policy subsidies to offset lost income. Furthermore, simultaneous participation in both PES programs and engagement in other livelihood activities, such as tourism, enhances household income, suggesting a synergistic effect. This study also reveals that household characteristics, such as livelihoods activities, household capital (i.e., house area and farmland area) and demographic factors (i.e., number of labor and non-labor members), exhibit spatial heterogeneity in their effects on household income across NRs. These findings highlight the importance of targeted, refined, and differentiated implementation plans or programs, as well as the consideration of interactions between policies and between policies and livelihood activities in policy design, implementation, and evaluation. These results and their implications are crucial for theoretical studies and evaluations on the design and implementation of conservation tools to achieve SDGs worldwide by scientists, policymakers, and conservation practitioners.

## Data availability statement

Data will be made available on request.

## CRediT authorship contribution statement

**Youqi Zhang:** Writing – original draft, Software, Methodology, Formal analysis, Data curation. **Yujun Wang:** Writing – original draft, Methodology, Investigation, Formal analysis, Data curation. **Hongbo Yang:** Software, Methodology, Investigation. **Vanessa Hull:** Writing – review & editing, Visualization. **Jindong Zhang:** Writing – review & editing, Writing – original draft, Software, Data curation, Conceptualization. **Fang Wang:** Data curation. **Zhiqiang Zhao:** Data curation. **Jianguo Liu:** Writing – review & editing, Project administration, Funding acquisition, Conceptualization.

## Declaration of competing interest

The authors declare that they have no known competing financial interests or personal relationships that could have appeared to influence the work reported in this paper.
