# Supplementary material for: Heterogeneous and interactive effects of payments for ecosystem services on household income across giant panda nature reserves
Source: Heliyon. 2024 Jul 18;10(15):e34866. doi: 10.1016/j.heliyon.2024.e34866 (PMC11320217; doi:10.1016/j.heliyon.2024.e34866)
Supplement: Multimedia component 1 [file mmc1.docx]

# **The sample questionnaire**

# ID: City County Town Village

# Date Investigator 。

# Comprehensive survey of social economy and natural environment of residents in the distribution area related to giant pandas conservation in 2015

The results of the survey will be for research purposes only, personal data is kept confidential, used anonymously and will not be provided to any organization. Thank you for your cooperation!

## Part I: Basic Family Information

1.1 Family information

| Number of populations |  | Farmland area (mu) |  |
| --- | --- | --- | --- |
| Number of adults |  | Grassland area (mu) |  |
| Number of students |  | Forest area (mu) |  |
| Number of elderly persons (> 65 years) |  | Family business |  |
| Number of people working in family farming (> 2 months) |  | Number of livestock | Pig: Sheep: Cattle: Horse:  Other: |
| Number of preschool children (< 6 years) |  | Number of poultry | Chicken: Duck: Other： |
| Number of migrant workers |  | Housing area (m²) |  |
| Net income at the end of the year |  | Type of housing structure | Masonry, wood, mixed, adobe houses |
| Total annual expenditure |  |  |  |
| GPS location | East Longitude: North Latitude: Elevation: | | |

1.2 Information on family members' migrant workers (including part-time jobs, work at local government and transportation operations)

| Member | Workplace | Working  months per year | Annual income (Yuan) | How much money providing to family | Other information |
| --- | --- | --- | --- | --- | --- |
|  |  |  |  |  |  |
|  |  |  |  |  |  |
|  |  |  |  |  |  |

**Part II: Economic Activities of Family Members**

2.1 Household expenditure

| Consumption items | | Amount | The location of the origin of the products |
| --- | --- | --- | --- |
| Food | Cooking oil |  |  |
|  | Salt, sauce, vinegar |  |  |
|  | Rice, noodles, flour |  |  |
|  | Vegetables, meat |  |  |
|  | Others (snacks, fruits, etc.) |  |  |
|  | Alcohol and cigarette |  |  |
| Educational costs | Student tuition fees |  |  |
|  | Living expenses (accommodation and food) |  |  |
|  | Transportation to and from home to school |  |  |
|  | Parents visit student expenses (including transportation expenses) |  |  |
|  | Other |  |  |
| Agricultural invest | Chemical fertilizer |  |  |
|  | Pesticide |  |  |
|  | Fodder |  |  |
| Energy | Natural gas |  |  |
|  | Coal |  |  |
|  | Electricity |  |  |
|  | Firewood |  |  |
|  | Other |  |  |
| Motor vehicle | Fuel oil |  |  |
|  | Insurance |  |  |
|  | Tolls |  |  |
|  | Maintenance and repair |  |  |
| Purchase of daily necessities (e.g., clothes, shoes and hats) | |  |  |
| Water | |  |  |
| Home appliances, furniture, etc. | |  |  |
| Medical expenses | |  |  |
| Repair of houses (including building materials and labor costs) | |  |  |
| Agricultural Tools | |  |  |
| Communication fees | |  |  |
| Public transport fees | |  |  |
| Loan / Interest | |  |  |
| Travel and entertainment | |  |  |
| Wedding and funeral gift money | |  |  |
| Other | |  |  |

2.2 Agricultural and animal husbandry income

| Categories | Varieties | Income (yuan) |
| --- | --- | --- |
| Grain | corn |  |
|  | potato |  |
|  | rice |  |
|  | wheat |  |
|  | other |  |
| Vegetable | turnip |  |
|  | vegetable |  |
|  | other |  |
| Fruit | orange |  |
|  | cherry |  |
|  | other |  |
| Livestock | honey |  |
|  | Pigs |  |
|  | horse |  |
|  | sheep |  |
|  | scalper |  |
|  | yak |  |
|  | poultry  (Chicken, duck) |  |
| Chinese herbal medicine | wild |  |
|  | planting |  |
| Others (e.g., tea, mushrooms) |  |  |

2.3 Agricultural produced costs ( for example, grain and vegetable seeds and fruit tree seeding etc. )

| Categories | Varieties | Cost(yuan) |
| --- | --- | --- |
| Grain | corn |  |
|  | potato |  |
|  | rice |  |
|  | wheat |  |
|  | other |  |
| Vegetable | turnip |  |
|  | vegetable |  |
|  | other |  |
| Fruit | orange |  |
|  | cherry |  |
|  | other |  |
| Cultivation | honey |  |
|  | Pigs |  |
|  | horse |  |
|  | sheep |  |
|  | scalper |  |
|  | yak |  |
|  | poultry  (Chicken, duck) |  |
| Chinese herbal medicine | wild |  |
|  | planting |  |
| Others (e.g., tea, mushrooms) |  |  |

2.4 Other income

|  | Income (yuan) | Cost (yuan) |
| --- | --- | --- |
| Rental income from Land and houses |  |  |
| Income from running restaurants, inns |  |  |
| Income from selling tourist souvenir |  |  |
| Income as guide |  |  |
| Income from Wedding and funeral gift money |  |  |
| Income from donation |  |  |
| Collecting bamboo shoot |  |  |
| others |  |  |

## Parts Ⅲ: relevant policies

1. To you family, how many cultivated land attend the GPGP program? And received how many subsidies?
2. Your family managed how many natural forests conservation areas and received how many subsidies?
3. Your agricultural technology information mainly comes from

A. Local government B. Scientific academies C. Non-profit organizations D. media E. neighbor F. other

1. Your protection policy information mainly comes from

A. Local government B. Scientific academies C. Non-profit organizations D. media E. neighbor F. other

1. Open question: What do you think is good for the future development of your village?？

A. tourism B. Planting C. livestock grazing D. other

What are your plans, ideas or suggestions for the future development of the industry?

*Thank you for participating!*
