# Supplementary material for: Heterogeneous and interactive effects of payments for ecosystem services on household income across giant panda nature reserves
Source: Heliyon. 2024 Jul 18;10(15):e34866. doi: 10.1016/j.heliyon.2024.e34866 (PMC11320217; doi:10.1016/j.heliyon.2024.e34866)
Supplement: Multimedia component 2 [file mmc2.docx]

**Table S1. Major characteristics of the 14 giant panda NRs included in this study**

| **Name** | **Abbreviation** | **Level** | **Mountain range** | **Number of wild pandas** | **Habitat area of wild pandas (hm^2^)** | **Human population around or in NRs** | **Average per capita annual income (yuan)** |
| --- | --- | --- | --- | --- | --- | --- | --- |
| Baihe | BH | Provincial | Minshan | 1 | 11321 | 6403 | 5439 |
| Baiyang | BY | Provincial | Minshan | 82 | 52408 | 7653 | 2458 |
| Baozuo | BZ | County-level | Minshan | 0 | 0 | 7901 | 1013 |
| Huanglong | HL | Provincial | Minshan | 15 | 19946 | 15569 | 9160 |
| Jiuzhaigou | JZG | National | Minshan | 3 | 24710 | 8667 | 1208 |
| Laojunshan | LJS | National | Liangshan | 3 | 3201 | 13889 | 6545 |
| Longdishui | LDS | County-level | Minshan | 13 | 20171 | 4743 | 1361 |
| Longxihongkou | LXHK | National | Minshan | 9 | 26707 | 9534 | 7886 |
| Meigu | MG | National | Liangshan | 22 | 35856 | 16320 | 1245 |
| Qianfoshan | QFS | National | Minshan | 12 | 11322 | 23368 | 1574 |
| Wanglang | WL | National | Minshan | 28 | 14727 | 4844 | 7316 |
| Wolong | WOL | National | Qionglai | 104 | 90458 | 4537 | 9826 |
| Xiaohegou | XHG | Provincial | Minshan | 49 | 25893 | 2726 | 1595 |
| Xiaozhaizigou | XZZG | National | Minshan | 47 | 40103 | 3784 | 3726 |

Note: The data comes from the Fourth Giant Panda Survey Report in Sichuan Province. 1 yuan equated to roughly 0.1592 USD in 2015.

**Table S2. Parameters in the model predicting household income**

| **Parameters** | **Description** | **Mean** | **SD** |
| --- | --- | --- | --- |
| **Household capital** | | | |
| House area | Family house area (m^2^) | 181.368 | 154.051 |
| Farmland | Household farmland area (Mu) | 4.7289 | 25.70046 |
| **Demographic factors** | | | |
| Non-labor | The sum of the number of elderly people over 65, students and children under 6 years old | 1.91 | 1.388 |
| Labor | Number of family laborers | 2.92 | 1.358 |
| **Livelihood activities** | | | |
| Migrant | Whether there are family members working outside (≥ 6 months) (1 yes; 0 no) | 0.29 | 0.455 |
| Tourism | Whether to participate in tourism related operations (1 yes; 0 no) | 0.13 | 0.341 |
| Agriculture | Whether to carry out agricultural production (1 yes; 0 no) | 0.37 | 0.484 |
| Graze | Is there any other grazing such as yaks? (1 yes; 0 no) | 0.13 | 0.341 |
| Other non-farm | Whether the family participates in other local livelihood activities besides the above livelihood activities (1 yes; 0 no) | 0.33 | 0.472 |
| **Policies participation** | | | |
| GTGP | Whether the family participates in the GTGP (1 yes; 0 no) | 0.85 | 0.358 |
| NFCP | Whether the family participates in the NFCP (1 yes; 0 no) | 0.41 | 0.492 |

**Table S3 . Interaction detector results**

|  | **Graphical representation** | **Interaction Result:** |
| --- | --- | --- |
| migrant ∩ tourism: | 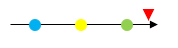 | Enhance, nonlinear- |
| migrant ∩ agriculture | 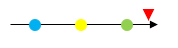 | Enhance, nonlinear- |
| migrant ∩ graze | 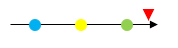 | Enhance, nonlinear- |
| migrant ∩ non-farm | 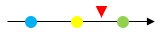 | Enhance, bi- |
| migrant ∩ GTGP | 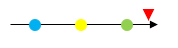 | Enhance, nonlinear- |
| migrant ∩ NFCP | 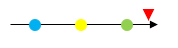 | Enhance, nonlinear- |
| tourism ∩ agriculture | 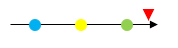 | Enhance, nonlinear- |
| tourism ∩ graze | 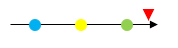 | Enhance, nonlinear- |
| tourism ∩ non-farm | 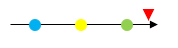 | Enhance, nonlinear- |
| tourism ∩ GTGP | 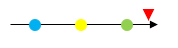 | Enhance, nonlinear- |
| tourism ∩ NFCP | 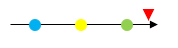 | Enhance, nonlinear- |
| agriculture ∩ graze | 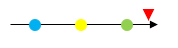 | Enhance, nonlinear- |
| agriculture ∩ non-farm | 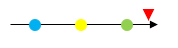 | Enhance, nonlinear- |
| agriculture ∩ GTGP | 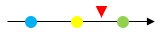 | Enhance, bi- |
| agriculture ∩ NFCP | 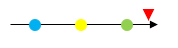 | Enhance, nonlinear- |
| graze ∩ non-farm | 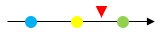 | Enhance, bi- |
| graze ∩ GTGP | 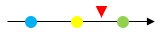 | Enhance, bi- |
| graze ∩ NFCP | 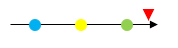 | Enhance, nonlinear- |
| non-farm ∩ GTGP | 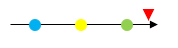 | Enhance, nonlinear- |
| non-farm ∩ NFCP | 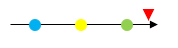 | Enhance, nonlinear- |
| GTGP ∩ NFCP | 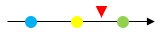 | Enhance, bi- |


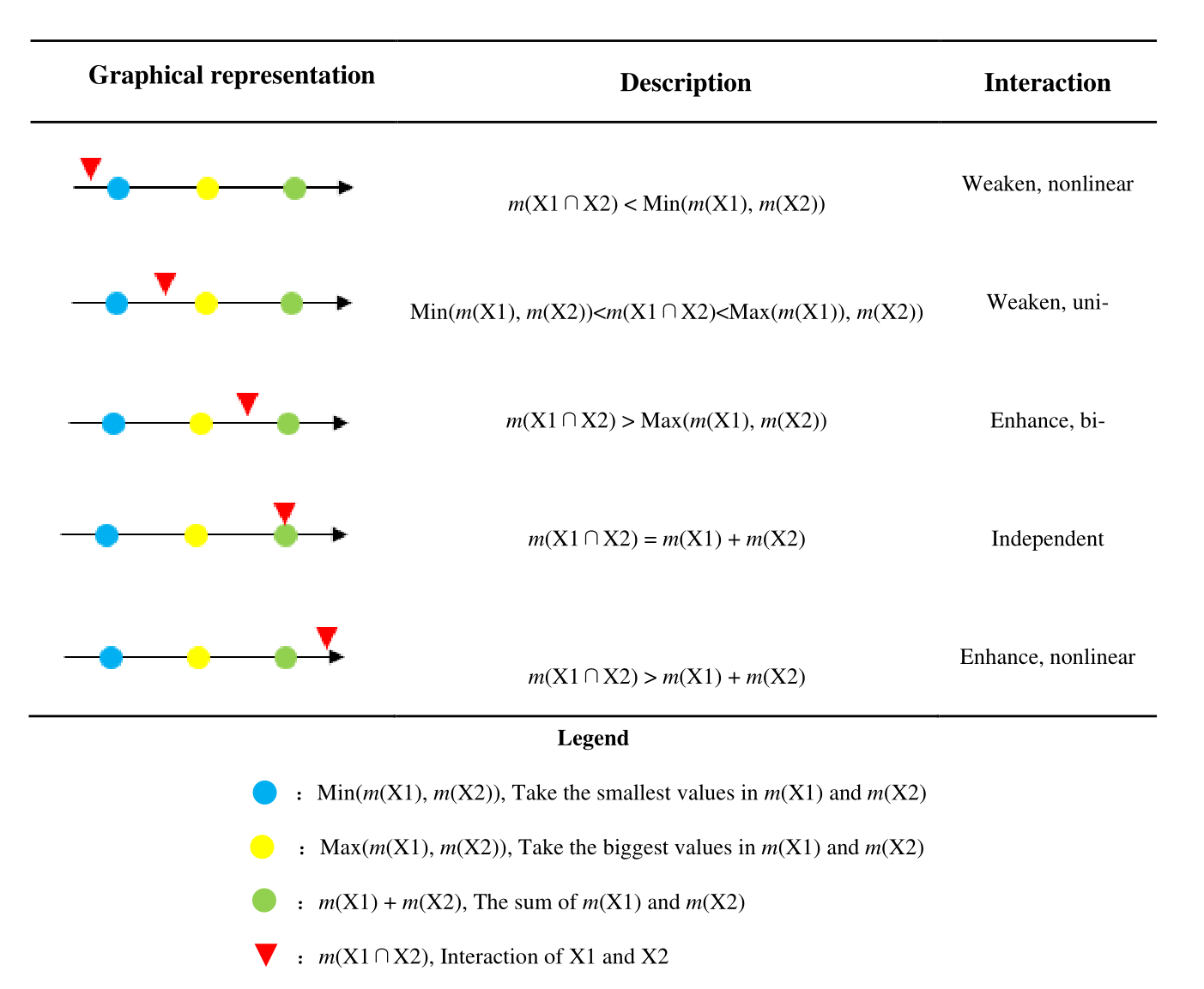


**Fig.S1. Types of interaction between variables**

（Reproduced from <http://www.geodetector.cn/>）
